# Crystal structure of ortho­rhom­bic {bis­[(pyridin-2-yl)meth­yl](3,5,5,5-tetra­chloro­pent­yl)amine-κ^3^ *N*,*N*′,*N*′′}chlorido­copper(II) perchlorate

**DOI:** 10.1107/S2056989015011792

**Published:** 2015-06-27

**Authors:** Katherine A. Bussey, Annie R. Cavalier, Jennifer R. Connell, Margaret E. Mraz, Kayode D. Oshin, Tomislav Pintauer, Danielle L. Gray, Sean Parkin

**Affiliations:** a Department of Chemistry & Physics, Saint Mary’s College, Notre Dame, IN 46556, USA; b Department of Chemistry & Biochemistry, Duquesne University, Pittsburgh, PA, 15282, USA; c School of Chemical Sciences, University of Illinois, Urbana-Champaign, IL 61801, USA; d Department of Chemistry, University of Kentucky, Lexington, KY 40506, USA

**Keywords:** crystal structure, four-coordinate copper(II), hetero-scorpionate complex, Atom Transfer Radical Addition (ATRA) reactions, disorder in cation and counter-ion

## Abstract

In the title compound, [CuCl(C_17_H_19_Cl_4_N_3_)]ClO_4_, the Cu^II^ ion adopts a distorted square-planar geometry defined by one chloride ligand and the three nitro­gen atoms from the bis­[(pyridin-2-yl)meth­yl](3,5,5,5-tetra­chloro­pent­yl)amine ligand. The perchlorate counter-ion is disordered over three sets of sites with refined occupancies 0.0634 (17), 0.221 (16) and 0.145 (7). In addition, the hetero-scorpionate arm of the bis­[(pyridin-2-yl)meth­yl](3,5,5,5-tetra­chloro­pent­yl)amine ligand is disordered over two sets of sites with refined occupancies 0.839 (2) and 0.161 (2). In the crystal, weak Cu⋯Cl inter­actions between symmetry-related mol­ecules create a dimerization with a chloride occupying the apical position of the square-pyramidal geometry typical of many copper(II) chloride hetero-scorpionate complexes.

## Abstract 2

In the crystal, weak Cu⋯Cl inter­actions between symmetry-related mol­ecules create a dimerization with a chloride occupying the apical position of the square-pyramidal geometry typical of many copper(II) chloride hetero-scorpionate complexes.

## Chemical context   

The mechanistic and structural study of Atom Transfer Radical Addition (ATRA) reactions is a growing and promising field in organometallic chemistry. These reactions involve the formation of carbon–carbon bonds through addition of a poly-halogenated saturated hydro­carbon to alkenes (Eckenhoff & Pintauer, 2010 ▸). Also known as the Kharasch reaction, most proceed either in the presence of a free-radical precursor as the halogen transfer agent, or a transition metal complex as the halogen transfer agent (Muñoz-Molina *et al.*, 2011 ▸). What makes these types of reactions attractive is generation of halogen-group functionalities within the product; which can be used as starting reagents in further functionalization reactions (Kleij *et al.*, 2000 ▸). Of inter­est to this project is analysis of hetero-scorpionate complexes incorporating weakly coordinating olefinic moieties in ATRA reactions. Since their discovery in the 1960s by Swiatoslaw Trofimenko (Pettinari, 2004 ▸), scorpionate ligands are considered to be some of the most useful ligand structures available in modern coordination chemistry (Trofimenko, 1999 ▸). As such, we report the synthesis and crystal structure of the title compound [Cu(C_17_H_19_N_3_Cl_4_)(Cl)][ClO_4_] (**1**).

## Structural commentary   

**Figure 1:**
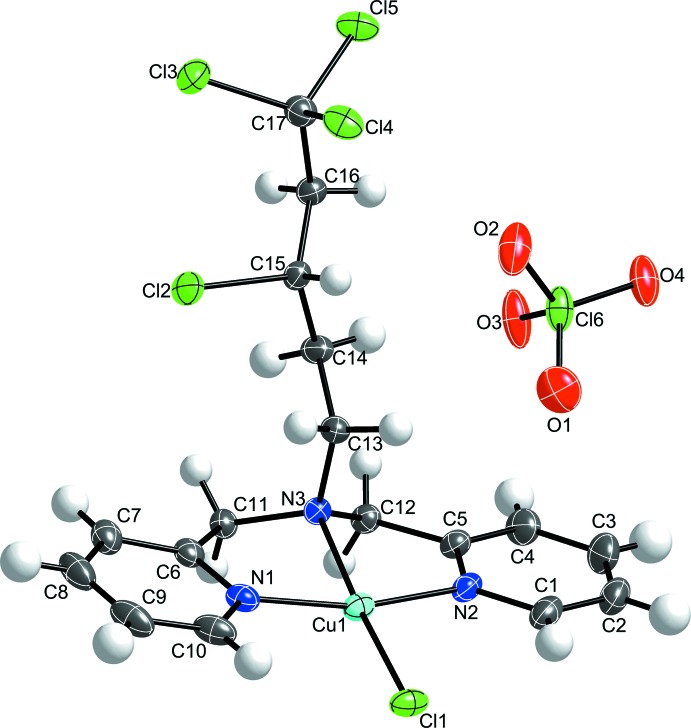
The mol­ecular structure of (**1**), shown with 50% probability ellipsoids for non-H atoms and circles of arbitrary size for H atoms. Only the primary orientations of the disordered sites are shown.

The title complex, (**1**) (Fig. 1 ▸), adopts a distorted square-planar geometry, as shown in the bond angles around the Cu^II^ ion. The Cu^II^ ion is coordinated by the binding of the two pyridine and amine nitro­gen atoms and a chlorido ligand. A τ-4 analysis of the distortions about the Cu^II^ ion yields a value of 0.15, slightly deviant from an ideal value of zero for perfect square-planar geometry [τ-4 = [360 – (α + β)]/141; Yang *et al.*, 2007 ▸] where α and β are the two greatest valence angles of the coordination center]. The Cu^II^ ion sits 0.0922 (4) Å out of the mean basal plane formed by Cl1 and the three coordinating N atoms, giving rise to the distortion from true square-planar geometry. The Cu—Cl1 [2.2519 (8) Å], Cu—N(amine) [2.027 (2) Å], and Cu—N(py) [1.982 (3) and 1.987 (3) Å] bond lengths are in the anti­cipated range for copper(II) complexes.
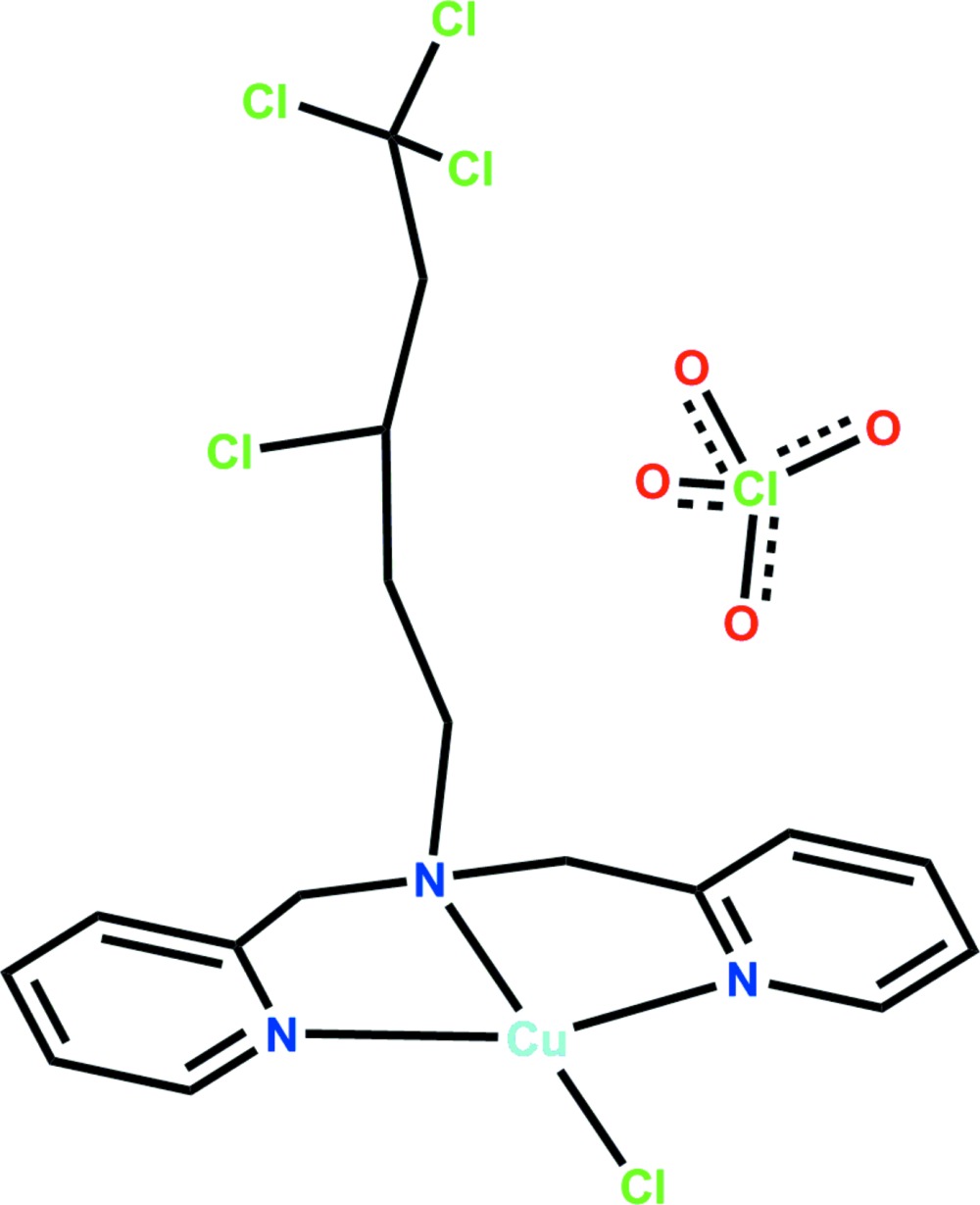


## Supra­molecular features   

**Figure 2:**
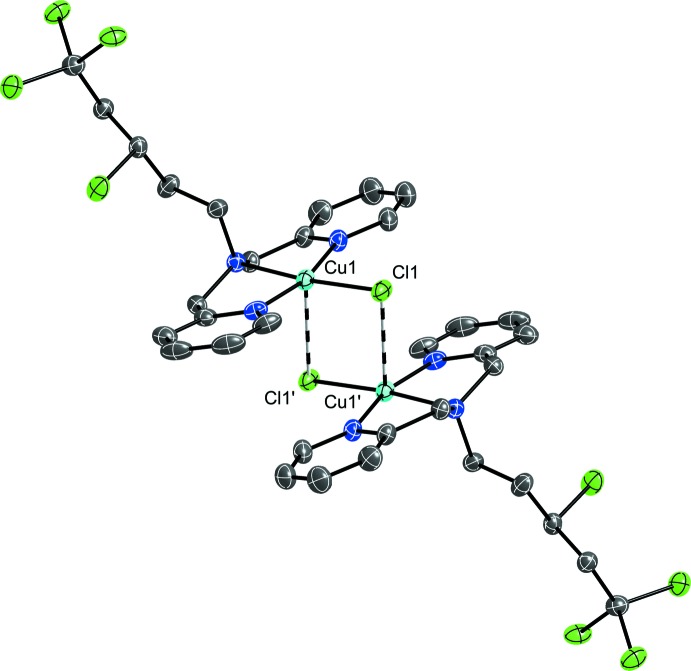
Dimer inter­actions between two [Cu(C_17_H_19_N_3_Cl_4_)(Cl)] (**1**) mol­ecules, shown with 50% probability ellipsoids for the primary orientations of the disordered sites. H atoms are removed for clarity. The symmetry operation to generate the additional cation is 1 − *x*, 1 − *y*, 1 − *z*.

**Figure 3:**
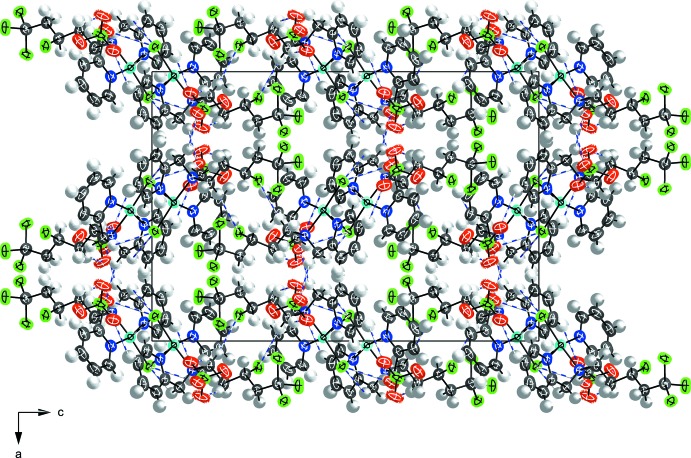
Packing diagram viewed along the *b*-axis direction showing the electrostatic inter­actions for the primary orientations of the disordered sites.

Weak [2.8535 (9) Å] Cu⋯Cl inter­actions between adjacent mol­ecules creates a dimerization with two Cl atoms bridging the Cu^II^ atoms (Fig. 2 ▸). The inter-copper distance between neighbouring cations is 3.4040 (7) Å. When considered, the weak Cu⋯Cl inter­action becomes the apical position of a distorted square-pyramidal geometry for the Cu^II^ atoms. Further strengthening the dimer are weak electrostatic C—H⋯ Cl inter­actions between C11—H11*A*⋯Cl1^i^ and C12—H12*B* ⋯Cl1^i^ (Cl1^i^ is generated by the symmetry operation − *x*, −*y* + 2, −*z*; Table 1 ▸). The three-dimensional packing structure (Fig. 3 ▸) is comprised from many weak C—H ⋯ O inter­actions that occur between carbon donors on the scorpionate arm or the bis­(pyridin-2-ylmeth­yl)amine and the oxygen atoms on varying orientations of the perchlorate counter-ion. Depending on the orientation of the chlorinated scorpionate arm, there are additional weak C—H⋯ Cl inter­actions.

## Database survey   

There are 200 structures with the bis­(pyridin-2-ylmeth­yl)amine ligand coordinating to copper with at least one bound chloride ligand (Groom & Allen 2014 ▸; CSD Version 5.36). Ignoring all the structures that have tethered pairs or tethered triplets of ligands, or have ligands whose amine group has substituents that additionally coordinate to the Cu^II^ atom, there are 58 remaining structures. Eighteen of these remaining structures have two bridging Cl ligands with one short axial Cu—Cl bond length (average 2.25 Å) and one long apical Cu—Cl bond length (average 2.72 Å).

## Synthesis and crystallization   

**Figure 4:**
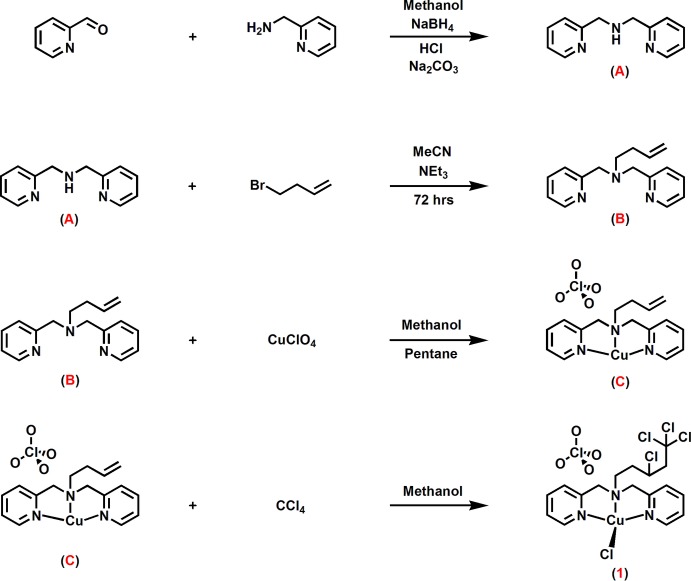
The synthetic scheme.

The synthetic procedure is outlined in Fig. 4 ▸. **Synthesis of 1-butene-** ***bis*** **(pyridin-2-ylmeth­yl)amine**, (**B**): bis­(pyridin-2-ylmeth­yl) amine (BPMA) precursor (**A**) was synthesized and purified following literature procedures (Carvalho *et al.*, 2006 ▸). BPMA (8.064 g, 40.5 mmol) was dissolved in 15 mL of aceto­nitrile followed by the addition of tri­ethyl­amine (4.098 g, 40.5 mmol) and 4-bromo­butene (5.468 g 40.5 mmol). The reaction was sealed and allowed to mix for 4 days to ensure complete deprotonation and coupling occurred. Generation of the tri­ethyl­amine hydrogen bromide salt Et_3_NH^+^·Br^−^ was observed as white crystals in the brown-colored solution. The mixture was filtered and the desired product extracted from the filtrate using a hexa­ne/water mixture. The hexane layer was separated and solvent removed to yield the ligand as a yellow-colored oil (8.516 g, 83%). The ligand was stored in a septum sealed round-bottom flask under argon gas in a refrigerator. ^1^H NMR (CDCl_3_, 400 MHz): δ2.31 (*dd*, *J* = 8.0 and 21.6 Hz, 2H), δ2.64 (*t*, *J* = 7.2 Hz, 2H), δ3.83 (*s*, 4H), δ4.97 (*d*, *J* = 10.4 Hz, 1H), δ5.01 (*d*, *J* = 18.8 Hz, 1H), δ5.75 (*m*, *J* = 10.4 Hz, 1H), δ7.13 (*t*, *J* = 6.4 Hz, 2H), δ 7.53 (*d*, *J* = 8.0 Hz, 2H), δ 7.64 (*t*, *J* = 7.6 Hz, 2H), δ8.51 (*d*, *J* = 4.4 Hz, 2H). ^13^C NMR (CDCl_3_, 400 MHz): δ 159.75, 149.01, 136.38, 135.38, 122.80, 121.88, 117.93, 77.13, 59.90, 57.32. FT–IR (liquid): *v* (cm^−1^) = 3066 (*w*), 2922 (*w*), 2816 (*w*), 2158 (*s*), 1639 (*s*), 1588 (*s*), 1361 (*s*), 994 (*w*), 756 (*s*). FT–IR (solid): *v* (cm^−1^) = 3394 (*w*), 3067 (*w*), 3008 (*s*), 2923 (*w*), 2817 (*s*), 2359 (*s*), 1619 (*s*), 1589 (*s*), 1432 (*s*).

**Synthesis of [Cu^I^(butene-** ***bis*** **(pyridin-2-ylmeth­yl)amine)][ClO_4_]**, (**C**): In the drybox, 1-butene-*bis*(pyridin-2-ylmeth­yl)amine (**A**) (1.00 g, 3.95 mmol) was dissolved in 5 mL aceto­nitrile in a 50 mL Schlenk flask. Cu(ClO_4_) (1.292 g, 3.95 mmol) was added to the flask to give a yellow-colored solution. The reaction was allowed to mix for 6 h, then 15 mL of pentane was slowly added to the solution to generate a yellow precipitate. Solvent was removed from the flask through a vacuum line. The precipitate was washed twice by transferring 10 mL of pentane into the flask and stirring vigorously for thirty minutes. Solvent was removed and the precipitate dried under vacuum for 2 h to yield a yellow-colored solid (2.109 g, 92%). ^1^H NMR (CD_3_CN, 400 MHz): δ2.45 (*dd*, *J* = 8.8 and 22.4 Hz, 2H), δ2.77 (*t*, *J* = 8.0 Hz, 2H), δ3.87 (*s*, 4H), δ4.92 (*d*, *J* = 10.0 Hz, 1H), δ4.98 (*d*, *J* = 16.8 Hz, 1H), δ5.70 (*m*, *J* = 10.4 Hz, 1H), δ7.33 (*d*, *J* = 8.0 Hz, 2H), δ 7.38 (*t*, *J* = 6.0 Hz, 2H), δ 7.80 (*t*, *J* = 7.6 Hz, 2H), δ 8.63 (*d*, *J* = 4.8 Hz, 2H). FT–IR (solid): *v* (cm^−1^) = 3271 (*w*), 3083 (*w*), 2923 (*w*), 2818 (*w*), 2325 (*s*), 2303 (*s*), 1602 (*s*), 1477 (*s*). TOF–ESI–MS: (*m/z*) [*M* – (ClO_4_)]^+^, Calculated for C_16_H_19_N_3_Cu 316.0875, found 316.0897 (7 p.p.m.).

**Synthesis of [Cu^II^(1,1,1-tri­chloro, 3-chloropentylbis(pyridin-2-ylmeth­yl)amine)][Cl][ClO_4_]**, (**1**): In the drybox, [Cu^I^(butene-*bis*(pyridin-2-ylmeth­yl)amine)][ClO_4_] (**C**) (0.50 g, 1.20 mmol) was dissolved in 5 mL aceto­nitrile in a glass vial with a stir bar. Nitro­gen gas purged CCl_4_ (0.174 mL, 1.80 mmol) was added to the vial producing a bluish-green-colored mixture. The reaction vial was sealed with a plastic cap and allowed to mix for 4 h, then removed from the drybox. Vapour diffusion crystallization at room temperature, incorporating 1 mL of the bluish-green solution with diethyl ether as the external diffusing solvent, produced blue-colored crystals suitable for X-ray analysis.

## Refinement   

Crystal data, data collection and structure refinement details are summarized in Table 2 ▸. The proposed structure model includes disorder of the hetero-scorpionate arm of the bis[(pyridin-2-yl)meth­yl](3,5,5,5-tetra­chloro­pent­yl)amine ligand over two sets of sites and disorder of the perchlorate anion modelled over three sites. The geometries of the disordered [C_5_H_7_Cl_4_] arm were restrained to be the same (s.u. 0.01Å). The perchlorate anions were also restrained to have the same geometries (s.u. 0.01 Å). In addition, the sum of the occupancies of the three orientations for the perchlorate anions were restrained to add up to one (s.u. 0.001). All disordered sites were restrained to have similar displacement amplitudes (s.u. 0.01) for atoms overlapping by less than the sum of van der Waals radii. Displacement parameters for the perchlorate anion positions were also restrained to behave relatively isotropic. All non-H atoms were refined with anisotropic displacement parameters. H atoms were included as riding idealized contributors, with C—H = 0.95 (aromatic), 0.99 (*sp* ^3^ C—*R*2H2), and 1.00 Å (*sp* ^3^ C—*R*3H). The *U*_iso_(H) values were set to 1.2*U*_eq_(C) of the carrier atom. The (002) reflection was omitted from the final refinement because it was partially obscured by the beam stop.

## Supplementary Material

Crystal structure: contains datablock(s) I. DOI: 10.1107/S2056989015011792/lh5771sup1.cif

Structure factors: contains datablock(s) I. DOI: 10.1107/S2056989015011792/lh5771Isup2.hkl

CCDC reference: 1407833

Additional supporting information:  crystallographic information; 3D view; checkCIF report

## Figures and Tables

**Table 1:** Hydrogen-bond geometry (, )

*D*H*A*	*D*H	H*A*	*D* *A*	*D*H*A*
C11H11*A*Cl1^i^	0.99	2.84	3.441(3)	120
C12H12*B*Cl1^i^	0.99	2.84	3.438(3)	120
C2H2O1^ii^	0.95	2.64	3.383(8)	136
C11H11*A*O4^iii^	0.99	2.53	3.123(7)	118
C12H12*A*O4	0.99	2.43	3.310(9)	148
C13H13*A*O1^iv^	0.99	2.64	3.578(11)	157
C14H14*B*O3^iv^	0.99	2.40	3.324(13)	154
C7H7O2^iii^	0.95	2.64	3.14(3)	113
C12H12*A*O4	0.99	2.37	3.32(2)	159
C14H14*C*O3^iv^	0.99	2.17	3.13(2)	163
C14H14*D*O4	0.99	2.41	3.26(2)	143
C16H16*D*O2^iv^	0.99	2.32	3.28(3)	162
C7H7O2"^iii^	0.95	2.57	3.26(2)	130
C12H12*A*O4"	0.99	2.53	3.33(4)	138
C13H13*A*O1"^iv^	0.99	2.45	3.33(2)	148
C2H2Cl4^v^	0.95	2.84	3.644(19)	143
C11H11*B*Cl2	0.99	2.70	3.490(7)	137
C13H13*B*Cl2^vi^	0.99	2.90	3.863(4)	165

Symmetry codes: (i) 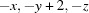; (ii) 
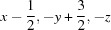
; (iii) 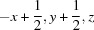; (iv) 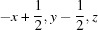; (v) 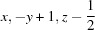; (vi) 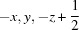.

**Table 2:** Experimental details

Crystal data	
Chemical formula	[CuCl(C_17_H_19_Cl_4_N_3_)]ClO_4_
*M*_r_	605.59
Crystal system, space group	Orthorhombic, *P* *b* *c* *n*
Temperature (K)	150
*a*, *b*, *c* ()	17.4845(13), 10.6593(8), 25.1030(18)
*V* (^3^)	4678.5(6)
*Z*	8
Radiation type	Mo *K*
(mm^1^)	1.65
Crystal size (mm)	0.72 0.31 0.04

Data collection	
Diffractometer	Bruker SMART APEXII CCD
Absorption correction	Multi-scan (*SADABS*; Bruker, 2014 ▸)
*T*_min_, *T*_max_	0.638, 0.746
No. of measured, independent and observed [*I* > 2(*I*)] reflections	51373, 6305, 4147
*R*_int_	0.081
(sin /)_max_ (^1^)	0.686

Refinement	
*R*[*F* ^2^ > 2(*F* ^2^)], *wR*(*F* ^2^), *S*	0.045, 0.108, 1.03
No. of reflections	6305
No. of parameters	437
No. of restraints	647
H-atom treatment	H-atom parameters constrained
_max_, _min_ (e ^3^)	0.56, 0.39

Computer programs: *APEX2*, *SAINT*, *XPREP* and *SADABS* (Bruker, 2014 ▸), *SHELXS2014/7* (Sheldrick, 2008 ▸), *SHELXL2014/7* (Sheldrick, 2015 ▸), *CrystalMaker* (CrystalMaker, 1994 ▸) and *publCIF* (Westrip, 2010 ▸).

## supplementary crystallographic information

### Crystal data

**Table:**

[CuCl(C_17_H_19_Cl_4_N_3_)]ClO_4_	*D*_x_ = 1.720 Mg m^−^^3^
*M**_r_* = 605.59	Mo *K*α radiation, λ = 0.71073 Å
Orthorhombic, *P**b**c**n*	Cell parameters from 5135 reflections
*a* = 17.4845 (13) Å	θ = 2.2–23.3°
*b* = 10.6593 (8) Å	µ = 1.65 mm^−^^1^
*c* = 25.1030 (18) Å	*T* = 150 K
*V* = 4678.5 (6) Å^3^	Prism, blue
*Z* = 8	0.72 × 0.31 × 0.04 mm
*F*(000) = 2440	

### Data collection

**Table:**

Bruker SMART APEXII CCD diffractometer	6305 independent reflections
Radiation source: fine-focus sealed tube	4147 reflections with *I* > 2σ(*I*)
Graphite monochromator	*R*_int_ = 0.081
profile data from φ and ω scans	θ_max_ = 29.2°, θ_min_ = 2.0°
Absorption correction: multi-scan (*SADABS*; Bruker, 2014)	*h* = −23→23
*T*_min_ = 0.638, *T*_max_ = 0.746	*k* = −14→14
51373 measured reflections	*l* = −34→34

### Refinement

**Table:**

Refinement on *F*^2^	647 restraints
Least-squares matrix: full	Hydrogen site location: inferred from neighbouring sites
*R*[*F*^2^ > 2σ(*F*^2^)] = 0.045	H-atom parameters constrained
*wR*(*F*^2^) = 0.108	*w* = 1/[σ^2^(*F*_o_^2^) + (0.0376*P*)^2^ + 5.7335*P*] where *P* = (*F*_o_^2^ + 2*F*_c_^2^)/3
*S* = 1.03	(Δ/σ)_max_ = 0.001
6305 reflections	Δρ_max_ = 0.56 e Å^−^^3^
437 parameters	Δρ_min_ = −0.39 e Å^−^^3^

### Special details

**Table:**

Geometry. All e.s.d.'s (except the e.s.d. in the dihedral angle between two l.s. planes) are estimated using the full covariance matrix. The cell e.s.d.'s are taken into account individually in the estimation of e.s.d.'s in distances, angles and torsion angles; correlations between e.s.d.'s in cell parameters are only used when they are defined by crystal symmetry. An approximate (isotropic) treatment of cell e.s.d.'s is used for estimating e.s.d.'s involving l.s. planes.

### Fractional atomic coordinates and isotropic or equivalent isotropic displacement parameters (Å^2^)

**Table:**

	*x*	*y*	*z*	*U*_iso_*/*U*_eq_	Occ. (<1)
Cu1	0.01473 (2)	0.91372 (4)	0.05612 (2)	0.02853 (11)	
N1	−0.01787 (15)	1.0476 (2)	0.10598 (10)	0.0314 (6)	
N2	0.06695 (16)	0.7705 (2)	0.02079 (10)	0.0307 (6)	
N3	0.10728 (14)	0.9061 (2)	0.10463 (10)	0.0255 (5)	
Cl1	−0.09392 (4)	0.90746 (8)	0.00802 (3)	0.03601 (19)	
C1	0.0368 (2)	0.6854 (3)	−0.01322 (13)	0.0409 (9)	
H1	−0.0161	0.6897	−0.0218	0.049*	
C2	0.0808 (3)	0.5933 (4)	−0.03554 (15)	0.0499 (10)	
H2	0.0586	0.5343	−0.0593	0.060*	
C3	0.1574 (3)	0.5865 (4)	−0.02351 (16)	0.0535 (11)	
H3	0.1884	0.5230	−0.0390	0.064*	
C4	0.1888 (2)	0.6722 (3)	0.01107 (15)	0.0439 (9)	
H4	0.2418	0.6693	0.0195	0.053*	
C5	0.14184 (18)	0.7632 (3)	0.03345 (12)	0.0301 (7)	
C6	0.03905 (18)	1.0893 (3)	0.13719 (12)	0.0299 (7)	
C7	0.0263 (2)	1.1791 (3)	0.17579 (14)	0.0394 (8)	
H7	0.0667	1.2054	0.1984	0.047*	
C8	−0.0459 (2)	1.2302 (3)	0.18115 (16)	0.0481 (10)	
H8	−0.0554	1.2931	0.2071	0.058*	
C9	−0.1039 (2)	1.1889 (4)	0.14842 (17)	0.0495 (10)	
H9	−0.1537	1.2238	0.1511	0.059*	
C10	−0.0885 (2)	1.0960 (4)	0.11176 (15)	0.0411 (9)	
H10	−0.1288	1.0654	0.0900	0.049*	
C11	0.11701 (18)	1.0358 (3)	0.12597 (13)	0.0286 (7)	
H11A	0.1441	1.0887	0.0996	0.034*	
H11B	0.1477	1.0337	0.1591	0.034*	
C12	0.17104 (17)	0.8613 (3)	0.07094 (12)	0.0283 (7)	
H12A	0.2119	0.8256	0.0936	0.034*	
H12B	0.1928	0.9323	0.0505	0.034*	
C13	0.08923 (19)	0.8171 (3)	0.14857 (12)	0.0313 (7)	
H13A	0.0711	0.7373	0.1328	0.038*	
H13B	0.0468	0.8522	0.1700	0.038*	
C14	0.1563 (2)	0.7886 (3)	0.18580 (13)	0.0380 (8)	
H14A	0.1893	0.8635	0.1894	0.046*	0.839 (2)
H14B	0.1875	0.7192	0.1711	0.046*	0.839 (2)
H14C	0.1674	0.6985	0.1800	0.046*	0.161 (2)
H14D	0.2001	0.8345	0.1702	0.046*	0.161 (2)
C15	0.1234 (2)	0.7506 (4)	0.24114 (14)	0.0278 (8)	0.839 (2)
H15	0.0768	0.6978	0.2358	0.033*	0.839 (2)
Cl2	0.09677 (7)	0.89437 (12)	0.27515 (4)	0.0431 (3)	0.839 (2)
C16	0.1823 (2)	0.6764 (4)	0.27201 (14)	0.0298 (8)	0.839 (2)
H16A	0.2273	0.7311	0.2777	0.036*	0.839 (2)
H16B	0.1991	0.6055	0.2494	0.036*	0.839 (2)
C17	0.1586 (2)	0.6238 (4)	0.32558 (16)	0.0352 (9)	0.839 (2)
Cl3	0.16533 (13)	0.73299 (18)	0.37901 (7)	0.0524 (5)	0.839 (2)
Cl4	0.06429 (15)	0.5634 (3)	0.32483 (14)	0.0415 (5)	0.839 (2)
Cl5	0.22215 (6)	0.49641 (11)	0.34156 (5)	0.0460 (3)	0.839 (2)
C15'	0.1645 (9)	0.8073 (10)	0.2458 (4)	0.032 (3)	0.161 (2)
H15'	0.2176	0.7933	0.2592	0.038*	0.161 (2)
Cl2'	0.1272 (4)	0.9613 (6)	0.2612 (2)	0.0464 (17)	0.161 (2)
C16'	0.1082 (9)	0.7056 (12)	0.2629 (5)	0.033 (3)	0.161 (2)
H16C	0.0566	0.7432	0.2661	0.040*	0.161 (2)
H16D	0.1058	0.6407	0.2347	0.040*	0.161 (2)
C17'	0.1283 (7)	0.6432 (11)	0.3150 (5)	0.040 (3)	0.161 (2)
Cl3'	0.1395 (7)	0.7557 (10)	0.3665 (4)	0.062 (3)	0.161 (2)
Cl4'	0.0539 (8)	0.5400 (16)	0.3321 (8)	0.049 (3)	0.161 (2)
Cl5'	0.2150 (4)	0.5561 (6)	0.3065 (3)	0.0541 (19)	0.161 (2)
Cl6	0.3626 (3)	0.9480 (5)	0.1331 (2)	0.0408 (9)	0.634 (17)
O1	0.4211 (4)	1.0019 (10)	0.1000 (3)	0.065 (2)	0.634 (17)
O2	0.3883 (6)	0.9535 (10)	0.1871 (3)	0.061 (2)	0.634 (17)
O3	0.2938 (5)	1.0211 (10)	0.1273 (5)	0.058 (2)	0.634 (17)
O4	0.3463 (6)	0.8219 (6)	0.1179 (4)	0.052 (2)	0.634 (17)
Cl6'	0.3611 (9)	0.9340 (15)	0.1444 (7)	0.051 (2)	0.221 (16)
O1'	0.4122 (12)	0.951 (2)	0.0995 (8)	0.059 (4)	0.221 (16)
O2'	0.4055 (14)	0.949 (3)	0.1915 (8)	0.056 (4)	0.221 (16)
O3'	0.2990 (12)	1.022 (2)	0.1436 (9)	0.051 (4)	0.221 (16)
O4'	0.3301 (11)	0.8105 (15)	0.1398 (10)	0.047 (4)	0.221 (16)
Cl6"	0.3552 (10)	0.9434 (19)	0.1397 (7)	0.052 (3)	0.145 (7)
O1"	0.4277 (11)	1.007 (2)	0.1349 (11)	0.061 (4)	0.145 (7)
O2"	0.3490 (14)	0.902 (2)	0.1936 (7)	0.059 (4)	0.145 (7)
O3"	0.2941 (15)	1.027 (4)	0.1259 (16)	0.053 (5)	0.145 (7)
O4"	0.356 (2)	0.843 (3)	0.1021 (10)	0.051 (4)	0.145 (7)

### Atomic displacement parameters (Å^2^)

**Table:**

	*U*^11^	*U*^22^	*U*^33^	*U*^12^	*U*^13^	*U*^23^
Cu1	0.02551 (19)	0.0343 (2)	0.02575 (19)	−0.00116 (17)	−0.00341 (16)	0.00344 (17)
N1	0.0324 (14)	0.0322 (14)	0.0296 (14)	0.0024 (12)	0.0030 (12)	0.0078 (11)
N2	0.0373 (15)	0.0336 (15)	0.0212 (13)	−0.0058 (12)	−0.0016 (11)	−0.0007 (11)
N3	0.0282 (13)	0.0250 (13)	0.0234 (12)	0.0014 (11)	−0.0012 (10)	−0.0007 (10)
Cl1	0.0270 (4)	0.0476 (5)	0.0334 (4)	−0.0072 (4)	−0.0093 (3)	0.0132 (4)
C1	0.053 (2)	0.042 (2)	0.0279 (17)	−0.0150 (17)	−0.0050 (16)	−0.0012 (15)
C2	0.076 (3)	0.043 (2)	0.0310 (19)	−0.006 (2)	−0.0020 (19)	−0.0086 (17)
C3	0.075 (3)	0.046 (2)	0.040 (2)	0.007 (2)	0.006 (2)	−0.0131 (18)
C4	0.047 (2)	0.046 (2)	0.039 (2)	0.0101 (18)	0.0055 (17)	−0.0053 (17)
C5	0.0344 (17)	0.0332 (17)	0.0227 (15)	−0.0004 (14)	−0.0009 (13)	−0.0001 (13)
C6	0.0364 (17)	0.0248 (15)	0.0284 (16)	0.0010 (14)	0.0046 (13)	0.0050 (13)
C7	0.053 (2)	0.0288 (17)	0.0361 (19)	−0.0016 (16)	0.0088 (17)	0.0013 (15)
C8	0.068 (3)	0.0301 (19)	0.046 (2)	0.0114 (18)	0.027 (2)	0.0071 (17)
C9	0.043 (2)	0.047 (2)	0.059 (3)	0.0165 (18)	0.023 (2)	0.017 (2)
C10	0.0326 (18)	0.047 (2)	0.044 (2)	0.0082 (16)	0.0095 (15)	0.0172 (18)
C11	0.0317 (16)	0.0243 (15)	0.0297 (16)	−0.0005 (13)	−0.0034 (13)	−0.0013 (13)
C12	0.0277 (16)	0.0302 (16)	0.0268 (16)	0.0015 (13)	−0.0011 (13)	−0.0007 (13)
C13	0.0423 (19)	0.0257 (16)	0.0258 (16)	0.0009 (14)	−0.0019 (14)	0.0022 (13)
C14	0.051 (2)	0.0356 (19)	0.0277 (17)	0.0136 (16)	−0.0045 (15)	−0.0004 (15)
C15	0.030 (2)	0.029 (2)	0.0240 (19)	0.0028 (16)	−0.0020 (16)	−0.0036 (15)
Cl2	0.0539 (7)	0.0435 (7)	0.0319 (6)	0.0184 (6)	0.0005 (5)	−0.0079 (5)
C16	0.0327 (19)	0.0306 (18)	0.0262 (17)	0.0009 (16)	−0.0005 (15)	0.0009 (15)
C17	0.038 (2)	0.033 (2)	0.034 (2)	0.0022 (18)	0.0023 (18)	0.0034 (17)
Cl3	0.0836 (14)	0.0450 (9)	0.0286 (8)	−0.0038 (8)	−0.0016 (7)	−0.0030 (6)
Cl4	0.0385 (8)	0.0382 (11)	0.0478 (11)	−0.0028 (7)	0.0107 (7)	0.0139 (8)
Cl5	0.0458 (6)	0.0461 (6)	0.0462 (7)	0.0073 (5)	−0.0033 (5)	0.0156 (5)
C15'	0.035 (5)	0.033 (5)	0.027 (5)	0.004 (5)	−0.004 (5)	−0.006 (5)
Cl2'	0.069 (4)	0.032 (3)	0.038 (3)	−0.006 (3)	−0.001 (3)	−0.002 (2)
C16'	0.039 (5)	0.033 (5)	0.027 (5)	0.000 (5)	0.001 (5)	−0.006 (5)
C17'	0.046 (5)	0.038 (5)	0.036 (5)	0.002 (5)	0.002 (5)	−0.003 (5)
Cl3'	0.082 (7)	0.061 (5)	0.042 (5)	−0.008 (5)	−0.003 (4)	−0.003 (4)
Cl4'	0.054 (6)	0.043 (6)	0.050 (6)	−0.009 (4)	0.012 (4)	0.019 (4)
Cl5'	0.052 (3)	0.054 (4)	0.057 (4)	0.011 (3)	−0.010 (3)	0.011 (3)
Cl6	0.0335 (13)	0.0350 (13)	0.0539 (17)	0.0105 (10)	−0.0121 (11)	−0.0163 (11)
O1	0.047 (3)	0.068 (5)	0.079 (4)	−0.007 (3)	−0.008 (3)	−0.002 (4)
O2	0.052 (5)	0.073 (4)	0.058 (3)	0.013 (3)	−0.031 (3)	−0.026 (3)
O3	0.039 (3)	0.039 (3)	0.095 (5)	0.013 (3)	−0.028 (3)	−0.029 (3)
O4	0.054 (4)	0.031 (3)	0.070 (5)	0.010 (3)	−0.020 (4)	−0.018 (3)
Cl6'	0.037 (3)	0.040 (3)	0.075 (4)	0.009 (3)	−0.021 (3)	−0.015 (3)
O1'	0.047 (6)	0.058 (7)	0.073 (6)	−0.001 (6)	0.007 (6)	−0.002 (6)
O2'	0.042 (7)	0.063 (7)	0.064 (7)	0.007 (6)	−0.023 (6)	−0.018 (6)
O3'	0.040 (6)	0.042 (6)	0.069 (8)	0.013 (5)	−0.032 (6)	−0.025 (6)
O4'	0.036 (6)	0.034 (6)	0.071 (7)	0.002 (5)	−0.015 (6)	−0.010 (6)
Cl6"	0.043 (4)	0.045 (4)	0.070 (4)	0.001 (4)	−0.018 (4)	−0.017 (4)
O1"	0.047 (6)	0.063 (7)	0.072 (7)	0.004 (6)	−0.016 (6)	−0.013 (7)
O2"	0.047 (7)	0.057 (7)	0.071 (7)	0.008 (6)	−0.012 (6)	−0.021 (6)
O3"	0.041 (8)	0.042 (8)	0.076 (9)	0.010 (8)	−0.021 (8)	−0.020 (8)
O4"	0.049 (7)	0.038 (7)	0.067 (8)	0.008 (7)	−0.013 (7)	−0.024 (7)

### Geometric parameters (Å, º)

**Table:**

Cu1—N1	1.982 (3)	C14—C15'	1.527 (10)
Cu1—N2	1.987 (3)	C14—C15	1.557 (5)
Cu1—N3	2.027 (2)	C14—H14A	0.9900
Cu1—Cl1	2.2519 (8)	C14—H14B	0.9900
N1—C6	1.342 (4)	C14—H14C	0.9900
N1—C10	1.346 (4)	C14—H14D	0.9900
N2—C5	1.350 (4)	C15—C16	1.512 (5)
N2—C1	1.353 (4)	C15—Cl2	1.815 (4)
N3—C12	1.479 (4)	C15—H15	1.0000
N3—C13	1.489 (4)	C16—C17	1.515 (5)
N3—C11	1.492 (4)	C16—H16A	0.9900
C1—C2	1.367 (5)	C16—H16B	0.9900
C1—H1	0.9500	C17—Cl4	1.771 (5)
C2—C3	1.375 (6)	C17—Cl3	1.780 (4)
C2—H2	0.9500	C17—Cl5	1.800 (4)
C3—C4	1.375 (5)	C15'—C16'	1.526 (10)
C3—H3	0.9500	C15'—Cl2'	1.807 (10)
C4—C5	1.390 (5)	C15'—H15'	1.0000
C4—H4	0.9500	C16'—C17'	1.509 (10)
C5—C12	1.496 (4)	C16'—H16C	0.9900
C6—C7	1.380 (4)	C16'—H16D	0.9900
C6—C11	1.504 (4)	C17'—Cl4'	1.758 (9)
C7—C8	1.381 (5)	C17'—Cl3'	1.774 (9)
C7—H7	0.9500	C17'—Cl5'	1.790 (10)
C8—C9	1.377 (6)	Cl6—O4	1.427 (4)
C8—H8	0.9500	Cl6—O2	1.429 (5)
C9—C10	1.378 (5)	Cl6—O1	1.438 (6)
C9—H9	0.9500	Cl6—O3	1.442 (4)
C10—H10	0.9500	Cl6'—O2'	1.424 (8)
C11—H11A	0.9900	Cl6'—O4'	1.429 (8)
C11—H11B	0.9900	Cl6'—O3'	1.434 (8)
C12—H12A	0.9900	Cl6'—O1'	1.449 (9)
C12—H12B	0.9900	Cl6"—O2"	1.427 (9)
C13—C14	1.530 (4)	Cl6"—O4"	1.429 (8)
C13—H13A	0.9900	Cl6"—O3"	1.436 (8)
C13—H13B	0.9900	Cl6"—O1"	1.444 (9)
			
N1—Cu1—N2	165.22 (11)	C15'—C14—C13	130.4 (6)
N1—Cu1—N3	83.05 (10)	C13—C14—C15	108.2 (3)
N2—Cu1—N3	82.56 (10)	C13—C14—H14A	110.0
N1—Cu1—Cl1	96.74 (8)	C15—C14—H14A	110.0
N2—Cu1—Cl1	97.22 (8)	C13—C14—H14B	110.0
N3—Cu1—Cl1	173.97 (8)	C15—C14—H14B	110.0
C6—N1—C10	119.3 (3)	H14A—C14—H14B	108.4
C6—N1—Cu1	113.2 (2)	C15'—C14—H14C	104.7
C10—N1—Cu1	127.5 (3)	C13—C14—H14C	104.7
C5—N2—C1	119.2 (3)	C15'—C14—H14D	104.7
C5—N2—Cu1	112.6 (2)	C13—C14—H14D	104.7
C1—N2—Cu1	128.2 (2)	H14C—C14—H14D	105.7
C12—N3—C13	112.2 (2)	C16—C15—C14	110.0 (3)
C12—N3—C11	114.7 (2)	C16—C15—Cl2	112.1 (3)
C13—N3—C11	110.4 (2)	C14—C15—Cl2	107.2 (3)
C12—N3—Cu1	105.74 (18)	C16—C15—H15	109.2
C13—N3—Cu1	107.54 (18)	C14—C15—H15	109.2
C11—N3—Cu1	105.65 (18)	Cl2—C15—H15	109.2
N2—C1—C2	121.4 (4)	C15—C16—C17	117.6 (3)
N2—C1—H1	119.3	C15—C16—H16A	107.9
C2—C1—H1	119.3	C17—C16—H16A	107.9
C1—C2—C3	119.8 (4)	C15—C16—H16B	107.9
C1—C2—H2	120.1	C17—C16—H16B	107.9
C3—C2—H2	120.1	H16A—C16—H16B	107.2
C2—C3—C4	119.5 (4)	C16—C17—Cl4	112.3 (3)
C2—C3—H3	120.2	C16—C17—Cl3	114.1 (3)
C4—C3—H3	120.2	Cl4—C17—Cl3	107.9 (2)
C3—C4—C5	118.9 (4)	C16—C17—Cl5	108.0 (3)
C3—C4—H4	120.6	Cl4—C17—Cl5	107.6 (2)
C5—C4—H4	120.6	Cl3—C17—Cl5	106.6 (2)
N2—C5—C4	121.2 (3)	C16'—C15'—C14	97.1 (8)
N2—C5—C12	116.0 (3)	C16'—C15'—Cl2'	110.7 (9)
C4—C5—C12	122.7 (3)	C14—C15'—Cl2'	107.2 (7)
N1—C6—C7	121.3 (3)	C16'—C15'—H15'	113.5
N1—C6—C11	115.9 (3)	C14—C15'—H15'	113.5
C7—C6—C11	122.8 (3)	Cl2'—C15'—H15'	113.5
C6—C7—C8	119.3 (4)	C17'—C16'—C15'	113.9 (9)
C6—C7—H7	120.3	C17'—C16'—H16C	108.8
C8—C7—H7	120.3	C15'—C16'—H16C	108.8
C9—C8—C7	119.2 (4)	C17'—C16'—H16D	108.8
C9—C8—H8	120.4	C15'—C16'—H16D	108.8
C7—C8—H8	120.4	H16C—C16'—H16D	107.7
C8—C9—C10	118.9 (3)	C16'—C17'—Cl4'	108.3 (9)
C8—C9—H9	120.5	C16'—C17'—Cl3'	111.0 (8)
C10—C9—H9	120.5	Cl4'—C17'—Cl3'	109.0 (9)
N1—C10—C9	121.8 (4)	C16'—C17'—Cl5'	108.9 (8)
N1—C10—H10	119.1	Cl4'—C17'—Cl5'	109.4 (8)
C9—C10—H10	119.1	Cl3'—C17'—Cl5'	110.2 (8)
N3—C11—C6	108.4 (2)	O4—Cl6—O2	110.8 (5)
N3—C11—H11A	110.0	O4—Cl6—O1	111.4 (4)
C6—C11—H11A	110.0	O2—Cl6—O1	108.0 (5)
N3—C11—H11B	110.0	O4—Cl6—O3	108.3 (4)
C6—C11—H11B	110.0	O2—Cl6—O3	109.7 (5)
H11A—C11—H11B	108.4	O1—Cl6—O3	108.6 (5)
N3—C12—C5	109.2 (2)	O2'—Cl6'—O4'	112.0 (10)
N3—C12—H12A	109.8	O2'—Cl6'—O3'	110.6 (10)
C5—C12—H12A	109.8	O4'—Cl6'—O3'	108.3 (10)
N3—C12—H12B	109.8	O2'—Cl6'—O1'	107.2 (10)
C5—C12—H12B	109.8	O4'—Cl6'—O1'	106.7 (10)
H12A—C12—H12B	108.3	O3'—Cl6'—O1'	111.9 (10)
N3—C13—C14	114.6 (3)	O2"—Cl6"—O4"	113.4 (12)
N3—C13—H13A	108.6	O2"—Cl6"—O3"	111.3 (12)
C14—C13—H13A	108.6	O4"—Cl6"—O3"	108.4 (11)
N3—C13—H13B	108.6	O2"—Cl6"—O1"	106.9 (11)
C14—C13—H13B	108.6	O4"—Cl6"—O1"	106.9 (11)
H13A—C13—H13B	107.6	O3"—Cl6"—O1"	109.8 (12)

### Hydrogen-bond geometry (Å, º)

**Table:**

*D*—H···*A*	*D*—H	H···*A*	*D*···*A*	*D*—H···*A*
C11—H11*A*···Cl1^i^	0.99	2.84	3.441 (3)	120
C12—H12*B*···Cl1^i^	0.99	2.84	3.438 (3)	120
C2—H2···O1^ii^	0.95	2.64	3.383 (8)	136
C11—H11*A*···O4^iii^	0.99	2.53	3.123 (7)	118
C12—H12*A*···O4	0.99	2.43	3.310 (9)	148
C13—H13*A*···O1^iv^	0.99	2.64	3.578 (11)	157
C14—H14*B*···O3^iv^	0.99	2.40	3.324 (13)	154
C7—H7···O2′^iii^	0.95	2.64	3.14 (3)	113
C12—H12*A*···O4′	0.99	2.37	3.32 (2)	159
C14—H14*C*···O3′^iv^	0.99	2.17	3.13 (2)	163
C14—H14*D*···O4′	0.99	2.41	3.26 (2)	143
C16′—H16*D*···O2′^iv^	0.99	2.32	3.28 (3)	162
C7—H7···O2"^iii^	0.95	2.57	3.26 (2)	130
C12—H12*A*···O4"	0.99	2.53	3.33 (4)	138
C13—H13*A*···O1"^iv^	0.99	2.45	3.33 (2)	148
C2—H2···Cl4′^v^	0.95	2.84	3.644 (19)	143
C11—H11*B*···Cl2′	0.99	2.70	3.490 (7)	137
C13—H13*B*···Cl2^vi^	0.99	2.90	3.863 (4)	165

Symmetry codes: (i) −*x*, −*y*+2, −*z*; (ii) *x*−1/2, −*y*+3/2, −*z*; (iii) −*x*+1/2, *y*+1/2, *z*; (iv) −*x*+1/2, *y*−1/2, *z*; (v) *x*, −*y*+1, *z*−1/2; (vi) −*x*, *y*, −*z*+1/2.
